# The Prevalence of Different Types of Headache in Patients with Subjective Tinnitus and Its Influence on Tinnitus Parameters: A Prospective Clinical Study

**DOI:** 10.3390/brainsci10110776

**Published:** 2020-10-24

**Authors:** Magdalena Nowaczewska, Michał Wiciński, Marcin Straburzyński, Wojciech Kaźmierczak

**Affiliations:** 1 Department of Otolaryngology, Head and Neck Surgery, and Laryngological Oncology, Ludwik, Rydygier Collegium Medicum in Bydgoszcz, Nicolaus Copernicus University, M. Skłodowskiej—Curie 9, 85-090 Bydgoszcz, Poland; 2 Department of Pharmacology and Therapeutics, Faculty of Medicine, Collegium Medicum in Bydgoszcz, Nicolaus Copernicus University, M. Skłodowskiej—Curie 9, 85-090 Bydgoszcz, Poland; wicinski4@wp.pl; 3 Headache Clinic—Terapia Neurologiczna Samodzielni, Maurycego Mochnackiego 10, 02-042 Warsaw, Poland; m.straburzynski@magazynorl.pl; 4 Department of Sensory Organs Examination, Faculty of Health Sciences, Collegium Medicum in Bydgoszcz, Nicolaus Copernicus University, M. Skłodowskiej—Curie 9, 85-090 Bydgoszcz, Poland; wojciech.kazmierczak@umk.pl

**Keywords:** tinnitus, tinnitus severity, headache, migraine, tension type headache, vertigo, THI, hearing loss, laterality

## Abstract

Both tinnitus and headache are very prevalent conditions in the general population, with bidirectional co-occurrence of them. A number of studies revealed a high prevalence of headache in tinnitus patients; however, most of them used self-reported symptoms, questionnaires, or health databases and were retrospective. The aim of this study was to evaluate the prevalence of different types of headache in a cohort of tinnitus patients and to assess the influence of headache on tinnitus parameters, focusing on appropriate headache and tinnitus diagnosis verified by clinical examination. This prospective study involved 286 patients diagnosed with subjective non-pulsating tinnitus. Patients’ clinical information was thoroughly assessed by the multidisciplinary team, including tinnitus characteristics and severity according to the Tinnitus Handicap Inventory (THI), loudness assessed by the Visual Analogue Scale (VAS), audiometry, type of headache diagnosed according to the third edition of the International Classification of Headache Disorders, severity of headache assessed by the Numeric Rating Scale (NRS), and impact of headache using the Headache Impact Test (HIT). In total, 141 (49.3%) tinnitus patients were diagnosed with headache, most of them with tension-type headache or migraine. They were significantly younger; mostly women; had bilateral tinnitus, vertigo, and depression more frequently; and had hearing loss less frequently as compared with the non-headache group. In total, 82 (58.16%) patients had the same localization of tinnitus and headache. Younger age, female gender, higher tinnitus burden measured by THI, and coexistence of hearing loss were independent variables connected with the occurrence of headache in the tinnitus group. According to our study, headaches impact tinnitus on many different levels and may be an important co-factor for tinnitus subtyping. We recommend screening for headache coexistence in all tinnitus patients.

## 1. Introduction

Tinnitus and headache are two very prevalent conditions in the general population [1,2,3,4]. Nevertheless, a link between those two disorders exists as headache was found to be more common in tinnitus patients than in the general population and tinnitus is more likely to be reported in patients with headache compared to the general population [5,6,7,8,9]. Tinnitus is the sensation of hearing a sound with no external auditory stimulus present, affecting up to 15% of the general population, diminishing quality of life, and placing a considerable burden on society due to financial repercussions of treatment cost [3,10]. Similarly, headache is a very prevalent symptom with a miscellaneous set of causes. According to the third edition of the International Classification of Headache Disorders (ICHD-3), we divide headache into two major groups: primary headaches (i.e., those without an underlying cause) and secondary headaches, which are linked with a specific etiology [2]. The most frequent type of primary headache is a tension-type headache (TTH) followed by migraine. As headache and tinnitus are very common in the population, the co-occurrence of them could be coincidental. However, previous studies revealed a high prevalence of headache in children and adults with tinnitus [11,12]. According to longitudinal studies, a history of migraine is a tinnitus risk factor [13,14,15]. On the other hand, it was found that 26.6% of patients with headache and 20% of migraine sufferers complain of tinnitus [6,16]. Another study revealed that the risks of tinnitus, sudden deafness and sensorineural hearing loss, were significantly higher in patients with non-migraine headache comparing with the non-headache group [17,18]. Migraine was found to have higher prevalence in patients with otolaryngological disorders than in the general population [19]. Additionally, the risk of cochlear disorders, especially for tinnitus, is significantly higher in individuals with a history of migraines [17]. In fact, brain–ear connections have been proposed as a reason for tinnitus and headache connection and their probable similar pathophysiological mechanisms [4,20,21,22,23]. It should be noted that in the majority of cases, unilateral headaches and unilateral tinnitus occur on the same side, thus alterations in trigeminal nerve activity have been suspected as a probable overlapping pathophysiological factor [24]. Other authors believe that tinnitus could be a symptom of allodynia associated with cortical hyperexcitability [25]. Additionally, headaches as a comorbidity have been proposed as a key co-factor for tinnitus subtyping [13].

It should be emphasized that all previous epidemiological studies regarding tinnitus and headache had one important limitation: the diagnosis of headache or/and tinnitus was based on self-reported headaches/tinnitus questionnaires or health insurance databases and were mostly retrospective [8,13,17,18,24]. Moreover, some of them considered only two types of headache: migraine and non-migraine headaches, and did not assess the severity and frequency of headache. Thus, the aim of this study was to evaluate the prevalence of different types of headache in a cohort of tinnitus patients and to assess the influence of headache on tinnitus parameters. Taking into consideration the limitations of previous studies, we focused on appropriate headache and tinnitus diagnosis to be verified by clinical examination, rather than being based on a questionnaire or self-reported.

## 2. Materials and Methods

### 2.1. Patients

This prospective study involved 286 patients recruited consecutively from the Department of Otolaryngology in University Hospital in Bydgoszcz, from February 2019 to May 2020. Patients were admitted to hospital (for scheduled diagnostic hospitalization) due to tinnitus and were finally diagnosed with subjective non-pulsating tinnitus. During hospitalization, all tinnitus patients were thoroughly assessed by a multidisciplinary team consisting of otorhinolaryngologists, a neurologist, and audiologists. Complete anamnesis as well as otological and neurological examination were applied to all tinnitus patients. They were asked about tinnitus onset and related clinical factors, the presence of comorbidities, and additional medical history. All patients were consulted by a neurologist specializing in headache. Other information about tinnitus was collected, including perceptional characteristics of the tinnitus sound, temporal properties (intermittent or continuous), location (in ear, or in the head, unilaterally or bilaterally), and severity. Patients were asked about the presence of vertigo and headache (the type of headache was diagnosed according to the International Classification of Headache Disorders (ICHD-3) [1]. Data about coexisting hypertension, diabetes, smoking, mood, sleep, and thyroid disorder were also collected. A routine blood test included vitamin D serum level, thyroid function tests, testosterone and estrogen levels, and lipid profiles. All tinnitus patients underwent carotid and vertebral Doppler ultrasonography with intima-media complex (IM complex) assessment. Most tinnitus patients underwent neuroimaging (computer tomography or magnetic resonance imaging), if it was necessary to set up diagnosis.

Exclusion criteria: pulsatile tinnitus, ear or brain surgery history, serious medical illness, serious chronic conditions (inflammatory, gastrointestinal, connective tissue, kidney and liver disorders), secondary headache due to cerebro-vascular diseases, idiopathic intracranial hypotension, Susac syndrome, and other causes.

All the procedures were approved by the Local Ethics Committee of the Ludwik Rydygier Collegium Medicum in Bydgoszcz (approval number KB 219/2019). Before the start of any procedure, all subjects gave their informed consent.

### 2.2. Tinnitus

The degree of perceived tinnitus severity was measured according to the validated Polish version of the Tinnitus Handicap Inventory (THI), while tinnitus loudness was assessed by the Visual Analogue Scale (VAS) for tinnitus loudness [26]. VAS scores were performed by asking the patient to rate the loudness of tinnitus from 0 to 10. THI evaluate the perceived severity of tinnitus and its impact on life. This scale is a survey with 25 items and divided into three subscales: functional, catastrophic, and emotional, with a total score ranging from 0 to 100. It grades five tinnitus severity categories: slight (0–16); mild (18–36); moderate (38–56); severe (58–76); and catastrophic (78–100). Psychoacoustic characteristics of tinnitus, including its loudness and pitch, were measured using the standard clinical method by presenting sounds similar to those described by the patient [27,28]. All patients underwent a familiarization procedure before the test. Audiometry was used to match tinnitus. Test frequencies were performed, included frequencies ranging from 250 to 16,000 Hz. The patients indicated the sound similar to their tinnitus after hearing sounds with different loudness and pitch. Patients with unilateral tinnitus received the test sound to the contralateral ear, while those with bilateral tinnitus to the ear with lower tinnitus loudness. In the cases of symmetrical tinnitus, the patients selected the ear to be tested themselves. The frequency and loudness of the sound were changed until the patients considered the sound to be similar to his/her tinnitus. Three measures of frequency and loudness each were performed, and the average of three repeated measurements was used in this process.

### 2.3. Headache

All patients were consulted by a neurologist specializing in headache. The type of headache was diagnosed according to the third edition of the International Classification of Headache Disorders (ICHD-3) [1]. We diagnosed headache as a disorder and excluded patients with symptomatic headaches. Other information about headache was collected, including location (unilateral, bilateral) and frequency. The severity of headache was assessed using the Numeric Rating Scale (NRS). The impact of headache was evaluated using the Headache Impact Test (HIT).

### 2.4. Audiometry

Pure tone audiometry was used to evaluate patients (in an acoustically treated booth) to test frequencies up to 16 kHz (audiometer, Interacoustics). Sensorineural hearing loss was defined according to WHO criteria, in the better ear, as an average of 500, 1000, 2000, and 4000 Hz [29]. If a patient had unilateral hearing loss, it was also defined as above. High-frequency hearing loss was defined as an average of 2000, 4000, 8000, and 16,000 Hz above 25 dB.

### 2.5. Statistics

Statistical analysis was performed using Microsoft Excel and RStudio software. Statistical significance was considered when *p* < 0.05. The Shapiro–Wilk test was used to assess the normality of each parameter’s distribution. For parameters on the nominal level, to evaluate the *p*-value, the chi square test or Fisher’s exact test was performed. For other parameters, differences between the means were calculated using a one-way ANOVA (analysis of variance) test (in the case of analyzing three groups) or a Student’s *t*-test (in the case of analyzing two groups). When the analyzed variable was not normally distributed, a Wilcoxon signed rank test was performed. Logistic regression was used to check the association of headache (dependent variable) with tinnitus in the tinnitus group. Independent variables, apart from basic data (age, gender), were related to tinnitus and hearing loss. We selected 12 independent variables. We used the area under the receiver operating characteristic (ROC) curves to measure the performance and the Hosmer–Lemeshow test to assess goodness of fit for the logistic regression model. 

## 3. Results

In total, 286 patients with subjective tinnitus were enrolled in this study and evaluated. In total, 141 (49.3%) tinnitus patients were diagnosed with headache. Patients with headache were significantly younger, were mostly women, and had mostly bilateral tinnitus. Additionally, in the headache group, the average hearing level of tinnitus matched at their tinnitus pitch had significantly lower loudness comparing with the non-headache group (39 ± 22 vs. 45 ± 22 dB). Patients with tinnitus and headache were diagnosed with hearing loss less frequently. In the non-headache group, tinnitus was mostly unilateral while in the headache group, there was a predominance of bilateral tinnitus. There were no statistically significant differences between groups regarding the duration and frequency of tinnitus, mean VAS and THI score, and vitamin D level. Patients with headache had more frequently coexistent vertigo than the non-headache group (73.76%) vs. 46.21%, *p* < 0.0001) as well as depression (32.62% vs. 20.69% *p* < 0.02). The characteristics of the tinnitus patients with and without headache are presented in Table 1.

Among the tinnitus with headache group, 42 (29.79%) patients were diagnosed with migraine, 94 (66.67%) with tension-type headache, and 21 (14.89%) with other types of headache. In total, 122 (86.52%) patients had only one type of headache, while 19 (13.48%) patients were diagnosed with two or more types of headache. All data about headache distribution are presented in Table 2.

Eighty-two (58.16%) patients had the same localization of tinnitus and headache, while 59 (41.84%) experienced tinnitus in different localizations as compared with headache side: 12 patients had bilateral tinnitus and left-side headache, 13 bilateral tinnitus and right-side headache, 23 left-side tinnitus and bilateral headache, and 11 right-sided tinnitus and bilateral headache. Interestingly, no patient had unilateral tinnitus and headache on the contralateral side. Patients with headache in the same localization as tinnitus did not significantly differ from patients with different localizations of headache and tinnitus regarding most of the variables (Supplementary Materials). We did not find any significant correlation between tinnitus parameters and headache in general. Only in chronic TTH patients was the duration of tinnitus significantly correlated with NRS scores (0.62, *p* < 0.084) and the presence of hearing loss negatively correlated with NRS (−0.58, *p* < 0.01).

Patient with tinnitus and migraine were significantly younger, and were mostly woman compared with the TTH group. There was a statistically significant difference regarding the severity of headache between the migraine and TTH group: migraine sufferers had higher scores in NRS and HIT scales accordingly (NRS 8.40 ± 0.89 vs. 5.55 ± 0.94, *p* < 0.0001; HIT 62.67 ± 7.50 vs. 50.20 ± 9.59, *p* < 0.0001). Similarly, migraine patients had higher scores in THI compared with TTH patients (accordingly 51 ± 29 vs. 40 ± 25, *p* < 0.0488). Patients with TTH tended to have high-frequency hearing loss more frequently and also high-density lipoprotein cholesterol (HDL|) and triglycerides blood levels were higher in this group compared with migraine patients. Women with migraine had significantly higher estradiol and testosterone levels compared with the TTH group. Moreover, migraine sufferers had lower IM complex and no plaques in carotid arteries. The differences between tinnitus patients with migraine or TTH are presented in Table 3.

Given the results presented in Table 2 and Table 3, we decided to go a step further and prepare a multivariate logistic model to evaluate factors connected with the presence of headache (dependent variable) in the tinnitus group. Independent variables, apart from basic data (age, gender), were related to tinnitus and hearing loss. Independent variables were selected from the database, including: vitamin D3 level (≤15, >15), gender (m/f), age (≤50, >50), mood disorders—at least one from the group sleep disorder, depression, anxiety (yes/no), vertigo (yes/no), diabetes (yes/no), headache (yes/no), and hearing loss (yes/no). From those factors (independent variables), an optimal set of parameters was selected to build a regression model. The process of selecting the optimal set of prognostic factors was performed using a backward selection procedure, starting with the model with all potential prognostic factors and eliminating irrelevant variables in subsequent steps. As a result of the analysis, three parameters were chosen. The *p*-values, odds ratios (ORs), and corresponding 95% confidence intervals (CIs) for selected parameters are presented in Table 4.

## 4. Discussion

Both tinnitus and headache are subjective, multifactorially influenced, and a very prevalent condition with considerable socioeconomic relevance. The present study aimed to access the prevalence of headache in tinnitus patients, focusing on appropriate headache diagnosis based on clinical examination. We diagnosed headache in almost 50% of tinnitus patients, which is a slightly higher percentage than reported by other authors. Pavaci et al. found headache in 45.38% of tinnitus patients, without information about headache type [30]. Langguth et al. revealed that about 27% of tinnitus patients suffered from headache, mostly migraine (45%), while only 13% of patients were diagnosed with TTH [13,24]. Contrary to their results, in our study, more tinnitus patients were diagnosed with TTH than migraine (66.67% vs. 29.79%). Similarly, Sindhusake et al. reported the prevalence of migraine in 32.6% of mild and 37.2% of severe tinnitus patients [31]. Another study found migraine in 25% of tinnitus sufferers [9]. Additionally, an association was found between migraine and tinnitus among young individuals, especially for migraine with aura [32]. In contrast, no relationship was found with a diagnosis of migraine, TTH, and tinnitus in a large cross-sectional study, where tinnitus was linked with increased cervical and pericranial muscle tenderness scores, rather than to any particular form of headache [16]. However, it should be emphasized that in our study, we diagnosed headache clinically, based on headache specialist consultation, while a majority of other studies used self-reported headache questionnaires or databases and were retrospective. Interestingly, 4.2% of our tinnitus patients were diagnosed with vestibular migraine, which is higher than data from the general population [33]. We also found 5.24% of patients had cervicogenic headache among the tinnitus group. Surprisingly, no patient was diagnosed with medication overuse headache or cluster headache, and only three with trigeminal or occipital neuralgia and only one with chronic migraine. The lack of severe types of headache in our cohort may have several explanations. On the one hand, chronic migraine or cluster headache sufferers may be bothered more by headache than tinnitus, thus they do not seek medical help regarding tinnitus. On the other hand, chronic and severe headache sufferers tend to overuse pain medication or may be on prophylactic treatment, which may protect them from developing tinnitus. Unfortunately, there are a lack of data in this area.

Regarding the tinnitus and headache relationship, a question arises regarding the pathophysiological pathways that may link these diseases. Shen et al. found the association between subclinical changes in cochlear function and TTH, as the TTH group had higher thresholds for both pure tone and extended high-frequency audiometry at most frequencies and all acoustic reflex thresholds were significantly higher in the TTH group than in controls [34]. Similarly, subclinical changes in auditory pathways and cochlear function were found to be associated with migraine [35,36]. Moreover, inner ear symptoms of phonophobia, hyperacusis, tinnitus, and fluctuation in hearing perception may be also migraine related, which indirectly indicates that cochlear blood vessels may be affected by basilar artery migraine. Vass described the trigeminal innervation of the inner ear vasculature: high levels of activity and sensory input produced rapid vasodilatatory responses of the inner ear [21]. Vass et al. also proposed that local plasma extravasation may be a result of excitation of the trigeminal nerve fibers in the cochlea, leading to the above inner ear symptoms [37]. They revealed a functional link between the cochlea and vertebro-basilar system by the capsaicin-sensitive primary sensory neurons [37]. Based on previous studies, clear evidence exists that changes in vascular permeability in the vertebrobasilar system, including blood supply to the cochlea and vestibule, may arise from electric or capsaicin stimulation of the trigeminal nerve.

It should be emphasized that headache (as pain condition) and tinnitus may have several clinical and pathophysiological similarities. In a very interesting set of publications, Møller found analogousness between chronic pain and tinnitus [22,38,39]. According to his papers, both pain and tinnitus have many different forms, have no physical signs, are phantom sensations, occur without any physical stimulation of sensory receptors, and may affect sleep and mood. Moreover, the severity of pain and tinnitus are difficult to assess quantitatively and depends on patients’ Additionally, perception and their perception is affected by many emotional and environmental factors. Furthermore, as tinnitus is often accompanied by hyperacusis, pain may be accompanied by allodynia, hyperpathia, and hypersensitivity. Regarding treatment, pain, tinnitus, and also headache can be modulated by electrical stimulation called neuromodulation [22]. Indeed, Volcy et al. hypothesized that spontaneous and aberrant neural activity may produce tinnitus in patients with headache, and tinnitus may be an allodynic symptom triggered by the trigeminal–cervical complex centralization and cerebral hyperexcitability [25].

Our tinnitus patients with headache were younger and predominantly woman compared with the non-headache group. Additionally, headache patients had significantly more frequent bilateral tinnitus that the non-headache group. Most patients had the same localization of headache and tinnitus and, significantly, no patient with unilateral tinnitus and unilateral headache had symptoms on the opposite sides. These findings are consistent with the study of Farri, who found bilateral tinnitus in 66% of patients with coexisting headache [40]. The similar location of tinnitus and headache raise the question about the pathophysiological link between them. For example, both tinnitus and migraine may have a genetic contribution, as bilateral tinnitus in men has been found to be significantly heritable compared with women, and young females with bilateral tinnitus also showed higher heritability than men [41]. According to another study, bilateral tinnitus was found to be influenced by genetic factors, which was not proven for unilateral tinnitus [42]. Thus, bilateral tinnitus may constitute a genetic subtype. Considering all data, one may ask if bilateral tinnitus is a separate and different tinnitus type? Song et al. examined patients with tinnitus and normal audiogram. They revealed the connection between bilateral tinnitus and hyperactivity at the level of the cochlear nucleus, as well as the involvement of the higher-order cortical area in unilateral tinnitus. Thus, the mechanism involved in the development of tinnitus may depend on its laterality [43]. Interestingly, it was also found that patients with unilateral tinnitus and normal hearing tended to have a higher THI score compared with bilateral tinnitus sufferers [43]. Tinnitus pitch, mean age of patients, and mean tinnitus duration were higher in the group with bilateral tinnitus [44]. Yang et al. reported that patients with bilateral tinnitus had more frequent and severe discomfort, were significantly older, and tended to have a longer duration of tinnitus than those with unilateral tinnitus [45]. Additionally, the THI and VAS scores were significantly higher in patients with bilateral than unilateral [45]. Moreover, patients with bilateral tinnitus had vertigo, dizziness, hyperacusis, and ear fullness more frequently than individuals with unilateral tinnitus [45].

Interestingly, the relationship between headache and tinnitus severity has been found, suggesting that headache may contribute to the distress caused by tinnitus as well as tinnitus distress contribute to headaches [8]. Moreover, in one study, the frequency of headaches was strongly correlated with the severity of tinnitus [46]. Volcy et al. described three tinnitus patients reporting an increase of tinnitus intensity only and consistently during headache exacerbations [25]. De Marinis et al. described a patient with a post herpetic neuralgia of the fifth cranial nerve and accompanying tinnitus located ipsilaterally to the painful side, which increased in proportion to the intensity of pain [47]. THI is the most common tool used for measuring the burden of tinnitus and defines the perceived impact of symptoms on the patient’s quality of life. In our study, there were no differences in tinnitus severity measured by the THI or NRS scales between the headache and non-headache group. However, patients with tinnitus and migraine had significantly higher THI compared with the non-headache group. Surprisingly, patients with tinnitus and headache had lower loudness of tinnitus comparing with no headache individuals. Nevertheless, the multivariate logistic model found that severe or catastrophic tinnitus burden (as measured with the THI scale) was an independent variable that is associated with the presence of headache in tinnitus patients. It is possible that both disorders have an additive effect on health-related quality of life and comorbid headaches may increase the amplification of sensory signals in tinnitus sufferers [13]. Additionally, tinnitus patients with coexisting headache had significantly less frequent unilateral hearing loss or high-frequency hearing loss as well as hypertension, hypertriglyceridemia, or carotid plaques, while they more often develop vertigo and depression. Thus, tinnitus patients without headache may have more underlying comorbidities, which act as tinnitus risk factors, especially dyslipidemia, arteriosclerosis, and impairment of the auditory system. On the other hand, a smaller number of tinnitus risk factors in the headache group may raise a question about the specific influence of headache itself on tinnitus appearance or its link with the younger age of patients in this group. Based on the differences between headache and non-headache tinnitus patients, it may be reasonable to include headache as a tinnitus subtyping factor.

In our study, the tinnitus patients with coexisting migraine had higher scores in THI compared with TTH individuals and the non-headache group and they also had high-frequency hearing loss, less frequently. Moreover, migraine patients had significantly higher scores on the NRS and HIT-6 scales. According to Sindhusake et al., migraine was associated with mild not severe tinnitus (RR 1.5.) [31]. Similarly, Dash et al. found that only 14% of migraine patients had documented hearing loss on pure tone audiometry [4].

A number of conditions link migraine and tinnitus. In vestibular migraine or basilar migraine, tinnitus may be one of the concomitant symptoms. Another interesting disorder connecting both is visual snow. This devastating neurological condition is associated with the continuous perception of tiny dots in entire visual field, resembling snow. Kondziela et al. found that the prevalence of visual snow syndrome is around 2% and revealed the association of this condition with tinnitus and headache, mostly migraine [48]. Another study not only confirmed this finding but also revealed that both tinnitus and migraine intensify visual snow by producing more additional visual symptoms [49]. This interaction between migraine, tinnitus, and visual snow indicates that all these conditions may share a common pathophysiologic mechanism connected with cortical disexcitability and thalamo-cortical dysrhythmia, both of which have also been link with migraine pathophysiology [49].

Up to 74% of headache patients complained of vertigo while in non-headache patients, only 46.21%. These results were confirmed by other studies. Langguth et al. found that patients with tinnitus and headache reported vertigo more frequently than the non-headache group [13]. Dash et al. revealed that 76% of migraine patients had vertigo on presentation, of which rotatory non-positional vertigo was the most common [4]. Bayazit et al. found dizziness in 30% of the migraine group, followed by 25% of vertigo, and 20% of tinnitus symptoms [6]. Interestingly, we found the same prevalence of vertigo in the migraine and TTH group. Contrary to our findings, Akdal et al. discovered that significantly more migraine than TTH sufferers experienced vestibular symptoms, like dizziness and vertigo [5]. It is possible that in our study, older age, higher prevalence of dyslipidemia, carotid artery arteriosclerosis, as well as concomitant tinnitus contributed to the higher than expected number of vertigo symptoms in TTH patients.

We revealed that depression was significantly more frequent in the tinnitus group with coexisting headache. Indeed, Boecking et al. found associations between tinnitus-related distress and pain perceptions. Tinnitus–pain associations may be influenced by psychological comorbidities, as they impact on depression, perceived stress, and coping attitudes [50].

Our study revealed a possible link between tinnitus and headache; however, it should be pointed out that data regarding this association are limited. There are no confirmed common pathways nor plausible rationale explaining the co-occurrence of these two disorders. Yet, tinnitus and migraine coexist in many individuals as shown by our study as well as some previous reports. Further studies are needed to better understand this association. Meanwhile, clinicians should look for and adequately manage both of these disorders in their patients.

We are aware of methodological limitations of this study as our data come from one center and may therefore not be representative. Our tinnitus patients were those more strongly affected by the disease, thus were more prone to seek medical help. Additionally, it should be emphasized that in our study, the prevalence of headache in tinnitus patients was not compared with a control group.

## 5. Conclusions

The prevalence of headache among tinnitus sufferers reaches almost half of cases. Tinnitus patients with coexisting headache differ from non-headache tinnitus cases, as they are younger, mostly woman, have a higher prevalence of bilateral tinnitus and vertigo, and have a lower coexistence of hearing loss. Younger age, female gender, higher tinnitus burden, and coexistence of hearing loss are independent variables associated with the occurrence of headache in the tinnitus group. According to our study, headaches impact tinnitus on many different levels and may be an important co-factor for tinnitus subtyping. All tinnitus patients should be screened for headache coexistence. More research should be done regarding tinnitus and headache to find if headache treatment may influence tinnitus frequency and severity.

## Figures and Tables

**Table 1:** Demographic and clinical characteristics of the tinnitus group, and depending on the presence of headache. Abbreviations THI: Tinnitus Handicap Inventory, VAS: Visual Analogue Scale; TTH: tension-type headache, TSH: thyroid-stimulating hormone, HDL: high-density lipoprotein cholesterol LDL: low-density lipoprotein cholesterol, IM complex: intima-media complex.

Parameters	Tinnitus Group without Headache *n* = 145	Tinnitus Group with Headache *n* = 141	*p*-Value
Age (mean ± SD)	54 ± 14	51 ± 14	0.0363
Gender (male/female)	77 (53.10%)/68 (46.90%)	42 (29.79%)/99 (70.21%)	<0.0001
Tinnitus bilateral, *n* (%)	56 (38.62%)	74 (52.48%)	0.0238
Tinnitus unilateral, *n* (%)	89 (61.38%)	67 (47.52%)	
Tinnitus in ear, *n* (%)	122 (84.14%)	117 (82.98%)	0.8735
Tinnitus in head, *n* (%)	23 (15.86%)	24 (17.02%)	
Tinnitus continuous, *n* (%)	119 (82.07%)	105 (74.47%)	0.1509
Tinnitus intermittent, *n* (%)	26 (17.93%)	36 (25.53%)	
Duration of tinnitus (years)	4.6 ± 5.01	5.1 ± 5.4	0.4583
VAS mean	6.1 ± 2.4	6.1 ± 2.7	0.9696
THI mean	38 ± 23	42 ± 26	0.1888
Tinnitus frequency (Hz)	3000 ± 2600	3000 ± 2900	0.9449
Tinnitus loudness (dB)	45 ± 22	39 ± 22	0.0189
Hearing loss unilateral, *n* (%)	71 (48.97%)	46 (32.62%)	0.0057
Hearing loss WHO, *n* (%)	32 (22.07%)	26 (18.44%)	0.4655
High frequency hearing loss, *n* (%)	87 (60.00%)	64 (45.39%)	0.0177
Vestibular disorders, *n* (%)	28 (19.31%),	21 (14.89%),	0.3493
Vertigo, *n* (%)	67 (46.21%)	104 (73,76%)	<0.0001
Vitamin D3 blood level, ng/dL	21.8 ±9.2	23.1 ±11.7	0.3535
Smoking, *n* (%)	13 (8.97%)	11 (7.80%)	0.8320
Depression, *n* (%)	30 (20.69%)	46 (32.62%)	0.0236
Sleep disorders, *n* (%)	33 (22.76%)	42 (29.79%)	0.1821
Anxiety, *n* (%)	11 (7.59%)	15 (10.64%)	0.4149
Hypertension, *n* (%)	51 (35.17%)	34 (24.11%)	0.05
Diabetes, *n* (%)	13 (8.97%)	8 (5.67%)	0.3660
Thyroid disorders, *n* (%)	30 (20.69%)	29 (20.57%)	1
Cholesterol, mg/dL	183 ±40	191 ±38	0.1148
Triglyceride, mg/dL	132 ±77	115 ±60	0.0307
HDL, mg/dL	52 ±14	55 ±16	0.1251
LDL, mg/dL	115 ±37	123 ±35	0.0399
Testosterone males, ng/dL	K: 31 ±12	K: 29.0 ±9.8	K: 0.3105
Testosterone females, ng/dL	M: 490 ±370	M: 450 ±190	M: 0.4559
Estradiol females, pg/mL	51 ±72	56 ±67	0.6310
TSH, mU/L	1.43 ±0.79	1.71 ±1.30	0.03035
Carotid plaques	35 (24.14%)	18 (12.77%)	0.0150
Intima media complex	0.91 ±0.28	0.91 ±0.29	0.9639

**Table 2:** The distribution of different types of headache in tinnitus patients.

Headache Type According to ICHD-3		*n*=	% of all Tinnitus Patients	% of all Headache Patients	The Same Localization of Tinnitus and Headache (*n*=)
All headaches		141	49.3%		82
Migraine	all	42	14.69%	29.79%	22
	With aura episodic	9	3.15%	6.38%	6
	Without aura episodic	32	11.19%	22.7%	16
	With aura chronic	1	0.35%	0.71%	0
	Without aura chronic	0	0	0	0
	Vestibular migraine	12	4.2%	8.51%	7
Tension-type headache	all	94	32.87%	66.67%	54
	Episodic	77	26.92%	54.61%	45
	Chronic	17	5.94%	12.06%	9
Other types of headache	all	21	7.34%	14.89%	14
	Cervicogenic	15	5.24%	10.64%	12
	Trigeminal neuralgia	1	0.35%	0.71%	1
	Occipital neuralgia	2	0.70%	1.41%	
	Post-traumatic	1	0.35%	0.71%	
	Frey syndrome	1	0.35%	0.71%	
	Primary stabbing	1	0.35%	0.71%	
All episodic headache		109	38.11%	77.3%	69
All chronic headache		17	5.94%	12.06%	9
1 type of headache		122	42,66%	86,52%	
2 types of headache		15	5.24%	10.64%	
3 types of headache		4	1.4%	2.84%	

**Table 3:** Demographic and clinical characteristics of the tinnitus group, depending on the type of headache.

Parameters *n*, % (Mean ± SD)	Tinnitus Group with Migraine *n* = 30 ^1^	Tinnitus Group with TTH *n* = 83 ^1^	*p*-Value
Age (mean ± SD)	44.5 ±9.9	51 ± 15	0.0064
Gender (male/female)	2 (6.67%)	31 (37.35%)	0.0010
	28 (93.33%)	52 (62.65%)	
Tinnitus bilateral, *n* (%)	16 (53.33%)	42 (50.60%)	0.8339
Tinnitus unilateral, *n* (%)	14 (46.67%)	41 (49.40%)	
Tinnitus in ear, *n* (%)	24 (80%)	69 (83.13%)	0.7813
Tinnitus in head, *n* (%)	6 (20%)	14 (16.87%)	
Tinnitus continuous, *n* (%)	20 (66.67%)	63 (75.90%)	0.3426
Tinnitus intermittent, *n* (%)	10(33.33%)	20(24.10%)	
VAS mean	3.6 ± 4.1	5.1 ± 5.4	0.1243
THI mean	51 ± 29	40 ± 25	0.0488
Frequency (Hz)	2300 ± 2200	3000 ± 2900	0.1713
Loudness (dB)	39 ± 21	39 ± 21	0.9635
HIT	62.67 ± 7.50	50.20 ± 9.59	<0.0001
NRS	8.40 ± 0.89	5.55 ± 0.94	<0.0001
Hearing loss unilateral *n* (%)	8 (26.67%)	8 (34.94%)	0.4989
Hearing loss WHO *n* (%)	3 (10%),	17 (20.48%),	0.2687
High frequency hearing loss, *n* (%)	9 (30%)	42 (50.60%)	0.0576
Vestibular disorders *n* (%)	4 (13.33%),	9 (10.84%)	0.7428
Vertigo *n* (%)	23 (76.67%)	62 (74.70%)	1
Vitamin D3 blood level ng/dL	23 ± 11	21.9 ± 9.9	0.7512
Smoking *n* (%)	2 (6.67%)	7 (8.43%)	1
Depression *n* (%)	7 (23.33%)	32 (38.55%)	0.1794
Sleep disorders *n* (%)	8 (26.67%)	24 (28.92%)	1
Anxiety *n* (%)	1 (3.33%)	10 (12.05%)	0.2829
Hypertension *n* (%)	4 (13.33%)	21 (25.30%)	0.2086
Diabetes *n* (%)	0 (0%)	4 (4.82%)	0.5719
Thyroid disorders *n* (%)	6 (20%)	17 (20.48%)	1
Cholesterol mg/dL	193 ± 41	189 ± 38	0.6524
Triglyceride mg/dL	89 ± 29	126 ± 69	0.0001
HDL mg/dL	63 ± 18	51 ± 15	0.0032
LDL mg/dL	124 ± 37	123 ± 35	0.9288
Testosterone (male) ng/dL	600 ± 170	430 ± 180	0.3900
Testosterone (female) ng/dL	32 ± 11	27.2 ± 9.0	0.0561
Estradiol (female) pg/mL	71 ± 74	40 ± 40	0.0524
TSH mU/L	2.01 ± 0.97	1.71 ± 1.52	0.2109
Carotid plaques	0 (0%)	12 (14.46%)	0.0340
Intima media complex	0.76 ± 0.16	0.96 ± 0.34	<0.0001

Abbreviations - THI: Tinnitus Handicap Inventory, VAS: Visual Analogue Scale; TTH: tension type headache, TSH: thyroid-stimulating hormone, HDL: high-density lipoprotein cholesterol LDL: low-density lipoprotein cholesterol, IM complex: intima-media complex. ^1^ Patients with coexistent migraine and TTH were excluded from this analysis.

**Table 4:** Multivariate logistic model evaluating independent variables connected with the presence of headache in tinnitus patients.

Multivariate Logistic Regression Model				
Parameter	OR	2.5% CI	97.5% CI	*p*-Value
THI (>57)	2.62	1.45	4.73	0.0013
Bilateral tinnitus	1.78	1.06	2.98	0.0291
Hearing loss (et least in one ear)	0.53	0.31	0.90	0.0182
Gender (men)	0.37	0.22	0.62	0.0001

## Supplementary Materials

The following are available online at https://www.mdpi.com/2076-3425/10/11/776/s1, Table S1. Demographic and clinical characteristics of tinnitus patients with headache, depending on the localization of tinnitus and headache.
