# Supplementary material for: The Prevalence of Different Types of Headache in Patients with Subjective Tinnitus and Its Influence on Tinnitus Parameters: A Prospective Clinical Study
Source: Brain Sci. 2020 Oct 24;10(11):776. doi: 10.3390/brainsci10110776 (PMC7694111; doi:10.3390/brainsci10110776)
Supplement: Supplementary file 1 [file brainsci-10-00776-s001.pdf]

Table S1. Demographic and clinical characteristics of tinnitus patients with headache, depending on the localization of tinnitus and headache.

Abbreviations - THI: Tinnitus Handicap Inventory, VAS: Visual Analogue Scale; TTH : tension type headache, TSH**:** thyroid-stimulating hormone, HDL: high-density lipoprotein cholesterol LDL: low-density lipoprotein cholesterol, IM complex: intima-media complex.

**Parameters**	**The same localisation of tinnitus and headache**<br>**n = 82**	**Different localisation of tinnitus and headache**<br>**n = 59**	p-value
Age (mean ± SD)	52 ±15	49 ±12	0.2090
Gender (male/female)	27 (32.93%)/ 55 (67.07%)	15 (25.43%)/44 (74.57%)	0.3575
Tinnitus bilateral, n (%)<br>Tinnitus unilateral, n (%)	49 (59.76%)<br>33 (40.24%)	25 (42.37%)<br>34 (57.63%)	**0.0597**
Tinnitus in ear, n (%)<br>Tinnitus in head, n (%)	71 (86.59%)<br>11 (13.41%)	46 (77.97%)<br>13 (22.03%)	0.2557
Tinnitus continuous, n (%)<br>Tinntus intermittent, n (%)	58 (70.73%)<br>24 (29.27%)	47 (79.66%)<br>12 (20.34%)	0.2475
Duration of tinnitus (years)	4.7 ±5.2	5.6 ±5.7	0.3551
VAS mean	6.2 ±2.8	6.0 ±2.6	0.6116
THI mean	42 ±26	42 ±27	0.8806
HIT	54.1 ±11.4	54.1 ±11.6	0.9975
NRS	6.4 ±1.5	6.5 ±1.6	0.4792
Tinnitus frequency (Hz)	3000 ±2600	2900±3200	0.9321
Tinnitus loudness (dB)	42 ±26	42 ±27	0.8806
Hearing loss unilateral, n (%)	31 (37.80%),	15 (25.42%),	0.14662
Hearing loss WHO, n (%)	16 (19.51%),	10 (16.94%),	0.8266
High frequency hearing loss, n (%)	41 (50%)	23 (38.98%)	0.2313
Vestibular disorders, n (%)	11 (13.41%),	10 (16.95%),	0.6344
Vertigo, n (%)	59 (71.95%)	45 (76.27%)	0.6984
Vitamin D3 blood level, ng/dl	22 ±11	24 ±13	0.5449
Smoking, n (%)	5(6.10%)	6(10.17%)	0.5263
Depression, n (%)	31 (37.80%)	15(25.42%)	0.1466
Sleep disorders, n (%)	27 (32.93%)	15(25.42%)	0.3575
Anxiety, n (%)	6 (7.32%)	9 (15.25%)	0.1686
Hypertension, n (%)	20 (24.39%)	14(23.73%)	1
Diabetes, n (%)	7 (8.54%)	1 (1.69%)	0.1389
Thyroid disorders, n (%)	14 (17.07%)	15 (25.42%)	0.2913
Cholesterol, mg/dl	188 ±38	195 ±37	0.2613

Triglyceride, mg/dl	117 ±58	111 ±63	0.5094
HDL, mg/dl	52 ±17	58 ±13	**0.0088**
LDL, mg/dl	123 ±34	124 ±36	0.8457
Testosteron males, ng/dl	450 ±180	460 ±220	0.8314
Testosteron females, ng/dl	27.1 ±9.1	31.3 ±10.3	**0.0367**
Estradiol females, pg/ml	44 ±54	72 ±78	**0.0474**
TSH, mU/l	1.68 ±0.93	1.75 ±1.70	0.7643
Carotid plaques	8 (9.76%)	10 (16.95%)	0.3061
Intima media complex	0.90 ±0.30	0.91 ±0.29	0.8115
